# Two-stage normalization using background intensities in cDNA microarray data

**DOI:** 10.1186/1471-2105-5-97

**Published:** 2004-07-21

**Authors:** Dankyu Yoon, Sung-Gon Yi, Ju-Han Kim, Taesung Park

**Affiliations:** 1 Program in Bioinformatics, Seoul National University, San56-l, Shin Lim-Dong, Kwan Ak-Ku, Seoul 151-747, Republic of Korea; 2 Department of Statistics, College of Natural Science, Seoul National University, San56-l, Shin Lim-Dong, Kwan Ak-Ku, Seoul 151-747, Republic of Korea; 3 SNUBI: Seoul National University Biomedical Informatics, Seoul National University School of Medicine, 28 Yongon-dong Chongno-gu, Seoul 110-799, Republic of Korea

## Abstract

**Background:**

In the microarray experiment, many undesirable systematic variations are commonly observed. Normalization is the process of removing such variation that affects the measured gene expression levels. Normalization plays an important role in the earlier stage of microarray data analysis. The subsequent analysis results are highly dependent on normalization. One major source of variation is the background intensities. Recently, some methods have been employed for correcting the background intensities. However, all these methods focus on defining signal intensities appropriately from foreground and background intensities in the image analysis. Although a number of normalization methods have been proposed, no systematic methods have been proposed using the background intensities in the normalization process.

**Results:**

In this paper, we propose a two-stage method adjusting for the effect of background intensities in the normalization process. The first stage fits a regression model to adjust for the effect of background intensities and the second stage applies the usual normalization method such as a nonlinear LOWESS method to the background-adjusted intensities. In order to carry out the two-stage normalization method, we consider nine different background measures and investigate their performances in normalization. The performance of two-stage normalization is compared to those of global median normalization as well as intensity dependent nonlinear LOWESS normalization. We use the variability among the replicated slides to compare performance of normalization methods.

**Conclusions:**

For the selected background measures, the proposed two-stage normalization method performs better than global or intensity dependent nonlinear LOWESS normalization method. Especially, when there is a strong relationship between the background intensity and the signal intensity, the proposed method performs much better. Regardless of background correction methods used in the image analysis, the proposed two-stage normalization method can be applicable as long as both signal intensity and background intensity are available.

## Background

cDNA microarrays consist of thousands of individual DNA sequences printed in a high density array on a glass slide. After being reverse-transcribed into cDNA and labeled using red (Cy5) and green (Cy3) fluorescent dyes, two target mRNA samples are hybridized with the arrayed DNA sequences or probes. Then, the relative abundance of these spotted DNA sequences can be measured. The ratio of the fluorescence intensity for each spot represents the relative abundance of the corresponding DNA sequence.

In cDNA microarray experiments, there are many sources of systematic variation. Normalization is the process of removing such variation that affects the measured gene expression levels. The main idea of normalization is to adjust for artifact differences in intensity of the two labels. Such differences result from differences in affinity of the two labels for DNA, differences in amounts of sample and label used, differences in photomultiplier tube and laser voltage settings and differences in photon emission response to laser excitation. Although normalization alone cannot control all systematic variations, normalization plays an important role in the earlier stage of microarray data analysis.

Many normalization methods have been proposed by using the statistical regression models. Kerr *et al. *[1] and Kerr *et al. *[2] suggested the ANOVA model approach. Wolfinger *et al. *[3] proposed a mixed effect model for normalization. Schadt *et al. *[4] proposed smoothing splines with generalized cross-validation (GCVSS). Kepler *et al. *[5] used a local polynomial regression to estimate the normalized expression levels as well as the expression level dependent error variance. Yang *et al. *[6] summarized a number of normalization methods for dual labeled microarrays such as global normalization and robust locally weighted scatter plot smoothing (LOWESS, Cleveland [7]). Workman *et al. *[8] proposed a robust nonlinear method for normalization using array signal distribution analysis and cubic splines. Wang *et al. *[9] suggested iterative normalization of cDNA microarray data to estimate normalization coefficients and to identify control gene set. Chen *et al. *[10] presented subset normalization to adjust for location biases combined with global normalization for intensity biases.

After performing two dye cDNA microarray experiments, we get foreground and background intensities from red channel and green channel, respectively. Although a complex modeling approach can be used, the signal intensity is usually computed by subtracting the background intensity from the foreground intensity. Thus, the noise in the background intensity may have a large effect on the signal intensity.

**Figure 1:**
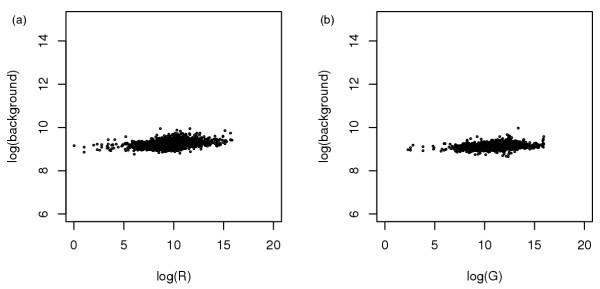
Example of correlations between background intensity and signal intensity from one of the NCI 60 data of Zhuo *et al. *16 (a) *log*(background) vs. *log*(*R*), correlation coefficient: 0.353, (b) *log*(background) vs. *log(G)*, correlation coefficient: 0.232.

Several approaches have been proposed for decreasing the background noises in image analyses (Yang *et al. *[11] and Kim *et al. *[12]). Kim *et al. *[13] found out the influences of background intensities on signal intensities, and showed that background intensities could play an important role in normalization. Recently, some background correction methods have been proposed using Bayesian method or smoothing function rather than simple subtraction when defining signal intensity (Kooperberg *et al. *[14] and Edwards [15]. As pointed out by Kim *et al. *[13], the signal intensities need to be robust to the local background intensity. In general, the signal intensities tend to have some correlations with background intensities (Figure 1). We think it is important to reduce variation in signal intensities caused by the background intensities. However, no systematic methods have been proposed that use the background intensities in normalization. In order to make the effect of background intensities more robust to the signal intensities, we propose a new method so called 'two-stage normalization method' to adjust for the effect of the background intensities. The first stage fits a regression model to adjust for the effect of background, and the second stage applies the usual normalization method such as a nonlinear LOWESS method to the background-adjusted intensities obtained from the first stage.

In order to perform the two-stage normalization method, we consider nine different background measures and investigate their performances in normalization. A detailed description on background measures is given in **Methods **section. Also, **Methods **section describes the proposed two-stage normalization methods. **Results **section describes the results from NCI 60 cDNA microarray experiment, which illustrates the effects of background intensities (Zhou *et al. *[16]). In addition, some comparative results are presented from cDNA microarrays of cortical stem cells of rat (Park *et al. *[17]) and those from kidney, liver, and testis cells from mice (Pritchard *et al. *[18]). The performance of two-stage normalization is compared to those of global normalization as well as intensity dependent nonlinear LOWESS normalization. We use the variability among the replicated slides to compare the performance of normalization methods. For certain selected background measures, the proposed two-stage normalization performs better than global or intensity dependent nonlinear normalization method. Finally, **Conclusion **section summarizes the concluding remarks.

## Methods

We propose a two-stage normalization method for the cDNA microarray data analysis using background intensities. At the first stage, we adjust for the effect of background intensities on *M*; at the second stage, we correct bias on *M *caused by other sources of systematic variation.

### Stage 1. Background normalization

Let *g*_*fi *_and *g*_*bi *_be the means(or medians) of the *i *th foreground and background intensity of green channel, respectively; *r*_*fi *_and *r*_*bi *_be the corresponding means(or medians) of red channel, respectively. Then for each spot, we have two pairs of intensities: (*g*_*fi*_, *g*_*bi*_) and (*r*_*fi*_, *r*_*bi*_), *i *= 1,..., *p*, where *p *is the number of spots in a slide. (For simplicity, we omit the subscript and define *A *and *M *using notation of Yang *et al. *[6] as follows:


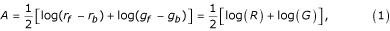


*M *= [log(*r*_*f *_- *r*_*b*_) - log(*g*_*f *_- *g*_*b*_)] = [log(R) - log(G)],     (2)

In cDNA microarray experiments, there are red and green background intensities. It would be desirable to consider the background intensities that are more closely related with the signal intensities. We consider nine possible background measures from red channel, green channel, and both channels as follows:

(a) Red channel

*Y*_*1 *_= log(*r*_*b*_),     (3)

*Y*_*2 *_= log(*r*_*f *_/ *r*_*b*_),     (4)

*Y*_*3 *_= log(*r*_*f*_)/log(*r*_*b*_),     (5)

(b) Green channel

*Y*_*4 *_= log(*g*_*b*_),     (6)

*Y*_*5 *_= log(*g*_*f *_/ *g*_*b*_),     (7)

*Y*_*6 *_= log(*g*_*f*_)/log(*g*_*b*_),     (8)

(c) Both channels


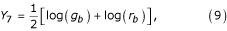



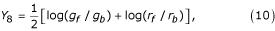



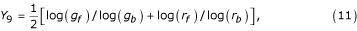


**Figure 2:**
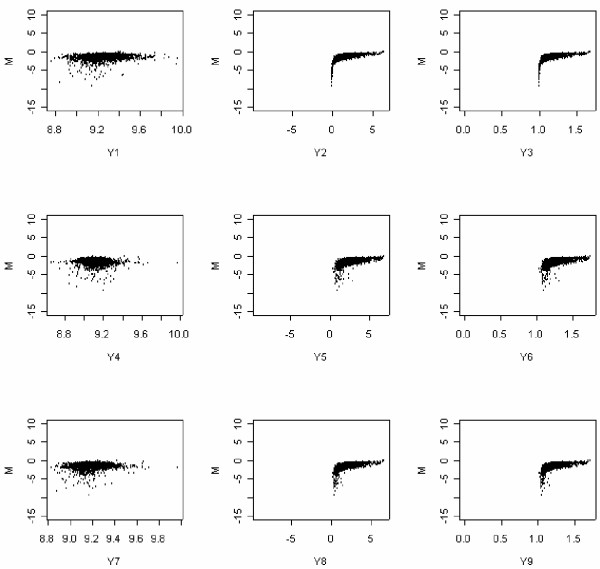
*M *versus *Y*_*k *_plots for NCI 60 data; the correlation coefficient of *M *= *log(R/G) *and *Y*_*k*_'s (*k *= l,...,9) are 0.2025, 0.6238, 0.6256, -0.0184, 0.5065, 0.5096, 0.1291, 0.5707, and 0.5729, respectively.

For each category, there are three types of background measures. The first one is log-transformed background intensities. The second one is the log-transformed ratio of foreground and background intensities. The third one is the ratio of the log-transformed values of foreground and background intensities. Here we used log base 2, but any logarithmic base can be used as desired. Figure 2 shows the relationship between signal intensities and these background measures. At the first stage, we adjust for the effect of background intensities by fitting the usual nonlinear LOWESS curve.

For simplicity, let *Y*_*k *_(*k *= 1,...,9) be an appropriate background intensity defined by red channel (a), green channel (b), or two channels (c). Then, fit the nonlinear LOWESS curve as follows:

*M*_*k *_= *C*^(1) ^(*Y*_*k*_),     (12)

*M*_*k*_^(1) ^= *M*_*k *_- *C*^(1) ^(*Y*_*k*_), for *k *= *1*,...,*9 *    (13)

where *C*^(1)^(·) represents the LOWESS curve and *M*_*k*_^(1) ^is the residual from the curve. Note that *M*_*k*_^(1) ^is the log-ratio of relative intensities after removing the effect of background intensities. For these ratios, we can perform the usual *MA *normalization at the second stage.

### Stage 2. *MA* normalization

In the second stage, we perform the normalization process as follows:

*M*_*k*_^(1) ^= *C*^(2) ^(*A*),     (14)

*M*_*k*_^(2) ^= *M*_*k*_^(1) ^- *C*^(2) ^(*A*),     (15)

where *C*^(2) ^(·) is the LOWESS curve and *M*_*k*_^(2) ^is the residuals from the curve in the second stage.

Note that at the second stage any normalization method can be applied including a simple global normalization method.

## Results

### Results of NCI 60 data

We first apply the proposed two-stage normalization method to a microarray data set of the NCI 60 cancer cell lines. These cell lines derived from human tumors have been widely used for investigations on various drugs and molecular targets . The National Cancer Institute's Developmental Therapeutic Programs  has been studying a large number of anti- cancer drug compounds and molecular targets on the 60 cancer cell lines (Weinstein *et al. *[19]). In particular, the NCI 60 microarray data have been frequently reanalyzed as an experimental model due to the inaccessibility to human tumor tissues for various studies on cancer. Using HCT116, one of the colon cell lines in the NCI 60 panel, Zhuo *et al. *[16] performed gene expression profile of dose- and time-dependent effects by the topoisomerase inhibitor I camptothecin compound (CPT). We here use a subset of the array data set consisting of ten slides. These slides were randomly selected to demonstrate the proposed method. Each slide contains 2,208 spotted clone sequences. We also apply global median normalization and intensity dependent nonlinear LOWESS normalization to this data set.

From ten slides we choose one slide to illustrate the proposed method. Figure 2 shows the plots of *M *versus *Y*_*k*_, where *M *= *log*(*R/G*) and *Y*_*k*_, *k *= 1,...,9 are background measures described in **Methods **section. The correlation coefficients between *M *and *Y*_*k*_'*s *(*k *= 1,...,9) are 0.2025, 0.6238, 0.6256, -0.0184, 0.5065, 0.5096, 0.1291, 0.5707, and 0.5729, respectively. Background measures *Y*_2 _and *Y*_3 _tend to have higher correlations than others.

**Figure 3:**
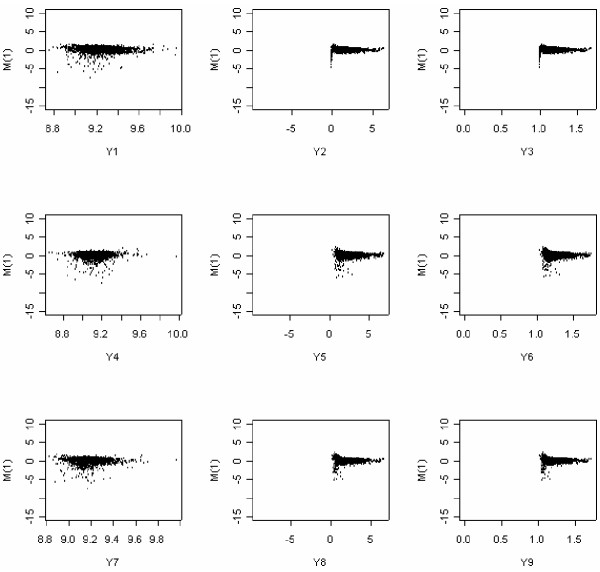
*M*^(1) ^versus *Y*_*k *_plot after the first stage normalization. Here, we can see reduction of bias for background intensity graphically (See Figure 2). Also, the correlation coefficient of each *Y*_*k *_decreased. The values are -0.0067, -0.0545, -0.0314, 0.2302, -0.0048, 0.0012, 0.0654, -0.0416, and -0.0297, respectively.

Figure 3 shows the results of the first stage normalization. The plots of *M*^(1) ^versus *Y*_*k*_, where *M*^(1) ^is the residual in equation (13) demonstrate some reduction in variability, which can be seen more clearly by comparing Figure 2 with Figure 3. Note that each correlation coefficient between *M*^(1) ^and *Y*_*k *_have values close to zero. Using *M*^*(1)*^, we carry out the second stage normalization.

**Figure 4:**
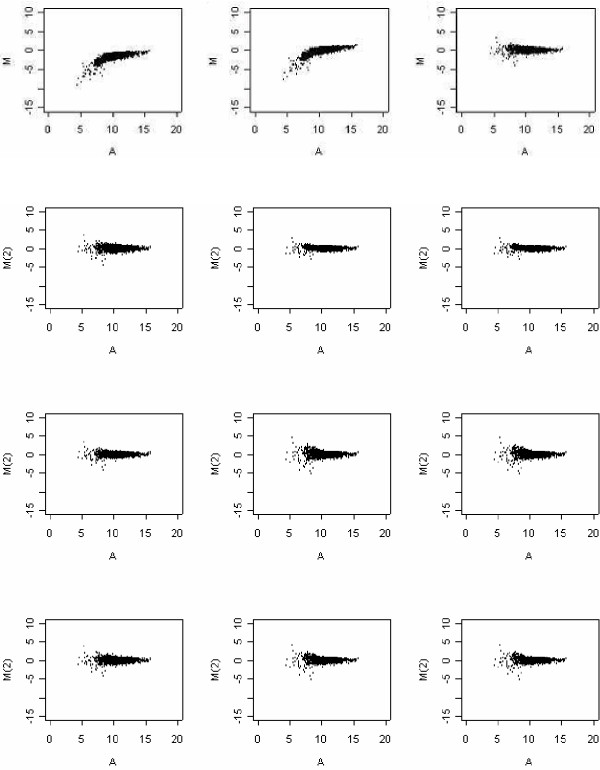
*MA *plots before and after normalization. The first row shows the *MA *plots for original (before normalization), after global normalization, and after nonlinear LOWESS normalization, respectively (from left to right), The second row shows *M*^*(2) *^versus *A *plots after two-stage normalization for *Y*_*1*_, *Y*_*2*_, and *Y*_*3 *_(from left to right), respectively. The third row shows *MA *plots for *Y*_*4*_, *Y*_*5*_, and *Y*_*6*_, respectively and the bottom row shows *MA *plots for *Y*_*7*_, *Y*_*8*_, and *Y*_*9*_, respectively. The two-stage normalization methods using *Y*_*2 *_and *Y*_*3 *_perform better than global and non-linear LOWESS normalization methods.

Figure 4 shows *MA *plots. The first row shows the *MA *plots for original (before normalization), after global normalization, and after nonlinear LOWESS normalization, respectively (from left to right), The second row shows *M*^(2) ^versus *A *plots after two-stage normalization for *Y*_1_, *Y*_2_, and *Y*_3_, respectively. The third row shows *MA *plots for *Y*_4_, *Y*_5_, and *Y*_6 _respectively, and the bottom row shows *MA *plots for *Y*_7_, *Y*_8_, and *Y*_9_, respectively. We can see that the two-stage normalization methods using *Y*_2 _and *Y*_3 _have the effect of reducing the variability among *Ms* and perform better than global and non-linear LOWESS normalization methods.

### Comparative studies

The goal of this study is to compare performances of normalization methods. We compare two-stage normalization to global median normalization and intensity dependent nonlinear LOWESS normalization. Following the idea of Park *et al. *[20], we use the variability among the replicated slides as comparison measures, which can be estimated by the mean square error (MSE). For each gene, we can calculate *MSE*_*l *_(*l *= 1,..., number of gene) which is the variance estimator for each gene derived from replicated slides. The main idea is that the better the normalization method, the smaller the variation among the replicated observations. Here, we use three different sets of microarray data: colorectal cancer data of NCI 60 (Zhou, *et al. *[16]), cortical stem cells data (Park, *et al. *[17]), and mouse gene expression data (Pritchard *et al. *[18]).

The goal of cortical stem cells study is to identify genes that are associated with neuronal differentiation of cortical stem cells. In this experiment, there are 3,840 genes in each slide from two experimental groups for comparison measured at six different time points (12 hrs, 1 day, 2 days, 3 days, 4 days, 5 days). All experiments were replicated three times, thus we have 36 slides for the analysis.

The objective of mouse gene expression study of Pritchard *et al. *[18] is to assess natural differences in murine gene expression. A 5406-clone spotted cDNA microarray was used to measure transcript levels in the kidney, liver, and testis from each of 6 normal male C57BL6 mice. Experiments were replicated four times per each mouse organ, two red fluorescent dye sample and two green dye samples. Since there are three organs, we have three sets of microarrays. In each organ, there are 24 slides available for the analysis.

In this comparative study, all five microarray data sets were used: colorectal cancer data set from NCI 60, cortical stem cells data set from Park *et al. *[17], and three organ data sets from Pritchard *et al. *[18]. Since results are similar among three organs, we only present the results of kidney. For simplicity, denote CCD for colorectal cancer data, SCD for stem cells data, and KD for kidney data, respectively.

**Figure 5:**
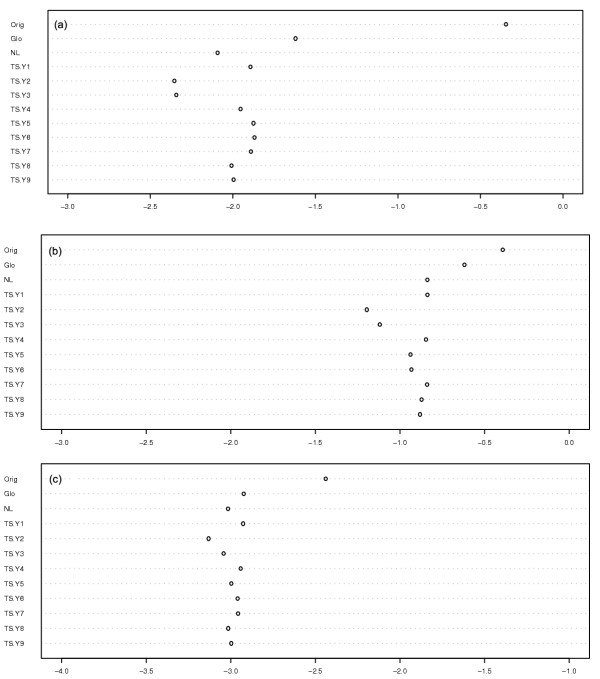
Dot plots of log-transformed variance estimates. *Y*-axis represents normalization methods and the *X*-axis represents the mean value of log-transformed variance estimates. (a) Colorectal cancer data set from NCI 60 (b) Cortical Stem Cells Data (Park *et al. *[17]) (c) Kidney data (Pritchard *et al.*[18]).

In this study, the performances of two-stage normalization using nine background measures are compared to global normalization and intensity dependent nonlinear LOWESS normalization. Figure 5 shows dot plots of log-transformed variance estimates for (a) CCD, (b) SCD, and (c) KD. Here each dot represents the mean value of the log-transformed MSEs for all genes. For all three different data sets, the global normalization reduces variability of the original data but the nonlinear LOWESS reduces variability much more. In general, these dot plots show that the two-stage normalization method using background intensities and the nonlinear normalization method have much smaller variabilities than those of global normalization. However, if we compare the two-stage normalization methods with the nonlinear normalization, the results differ depending on the background measures. That is, the background measures *Y*_2 _and *Y*_3 _in two-stage normalization methods always yield better performances than the nonlinear normalization method, while the other background measures yield comparable results to those of the nonlinear normalization. Thus, we suggest either *Y*_2 _or *Y*_3 _as background measures in the two-stage normalization.

## Conclusions

In microarray studies, many undesirable systematic variations are commonly observed. Normalization becomes a standard process for removing some of the variation that affects the measured gene expression levels. One major source of variation is the background intensities. Recently, some methods have been employed for adjusting for the background intensities. However, all these methods focus on defining signal intensities appropriately from foreground and background intensities during the image analysis (Kooperberg *et al. *[14], Edwards [15]). Although a number of normalization methods have been proposed, no systematic methods have been proposed using the background intensities in the normalization process.

In this paper, we propose two-stage normalization for adjusting for the effect of background intensities systematically. The motivating idea is that the noise caused by background intensities may increase the variability in signal intensities. Although we use the log-transformed ratio of two channels denoted by *M *in most subsequent analysis, the noise caused by background intensities may still remain in *M *even after normalization. The two-stage normalization may be quite effective especially when there is a high correlation between *M *and background measures such as *Y*_2 _and *Y*_3_*.*

Among nine background measures, we recommend two background measures *Y*_2 _and *Y*_3 _based on the results of the comparative studies. For these two background measures, we show that the two-stage normalization method always performs better than the global normalization methods and the nonlinear LOWESS normalization method.

We wondered if the relative good performance of two-stage normalization using *Y*_2 _and *Y*_3 _is due to low intensities. We investigated this problem for NCI 60 data after removing spots with low intensities. The spots whose ratio of foreground and background intensities were smaller than 1.5 were removed in the analysis. This new data set also provided quite similar results.

The main reasons why background measures *Y*_2 _and *Y*_3 _perform better than other backgrounds are as follows. The background fluorescence might be relatively strong in the Cy5 channel due to interaction between the slide substrate and the hybridization materials. This effect is weaker in the Cy3 channel. It might be also possible that the background fluorescence in the Cy5 channel inflates the values of *r*_*b *_without correspondingly inflating the values of *r*_*f*_. This means that for weakly-responding spots, the *r*_*f *_and *r*_*b *_values are similar. This produces very low values in *M*, *Y*_2 _and *Y*_3 _for these spots. Note values under 5 for *log(R) *but not for *log(G) *in Figure 1. Also note downward outliers but not upward outliers for *M *in all panes in Figure 2. These artefacts in *M *are partially corrected by regressing against *Y*_2 _or *Y*_3_*. *However, the effectiveness of the proposed method for other data may depend on the background fluorescence artefacts.

The comparison is based on the variability measures derived from the replicated microarray samples. These variability measures can be easily derived from any replicated microarray experiment. Although we have studied only a limited number of data sets, our findings are quite consistent.

For these data sets, we also conduct a similar analysis to see the effect of print-tips in normalization process. The results were consistent to those in Figure 5 and not reported here. Background measures *Y*_2 _and *Y*_3 _yielded better results than other background measures.

**Figure 6:**
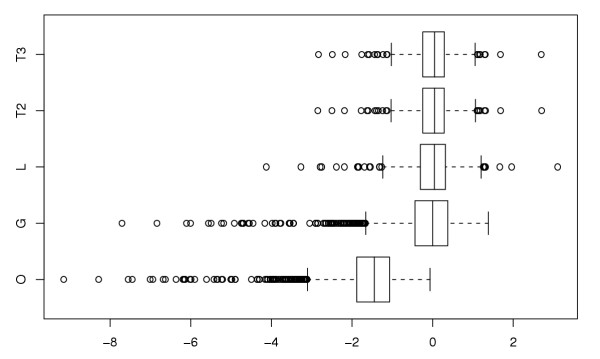
Density plots of *M* for one of the colorectal cancer data set from NCI 60. The original one(O), after the global normalization(G), after LOWESS normalization(L), and two-stage normalization using *Y2* and *Y3* (T2 and T3, respectively).

One major concern for normalization is about over-fitting which may cause overcorrection of real biological significance. In fact, every normalization method has a possibility of overcorrecting the data and removing some existing biological significance in the data. For the NCI60 data, for example, it is not easy to find out whether such negatively low expression genes are biologically significant genes or not. We investigate the possibility of overcorrecting by drawing the density plot of *M*: the original one (O), after the global normalization (G), after LOWESS normalization (L), and two-stage normalization using *Y2* and *Y3* (T2 and T3, respectively). The density plot is in Figure 6. The distributions of *M* for T2 and T3 are quite similar to that of L. This simple empirical investigation shows that the proposed method does not always cause a bigger overcorrection than the LOWESS normalization. However, we must be careful for over-fitting which may overcorrects the biological findings of interest.

In addition, we performed the hypothesis test for M equal to zero and counted the number of genes that have different expression level between two channels. The number of significant gene with different expression value is 909 for the original data before normalization, 326 for the data after global normalization, 302 for the data set after LOWESS normalization, and 297 for the data after two-stage normalization. The numbers of significant genes do not differ much among normalization methods.

The proposed method can be applicable when both background and foreground intensities are available after the image analysis. Many image software provides both signal intensity as well as background intensity of spots. In most cases, the signal intensity is defined by simple subtraction such as (*r*_*f *_- *r*_*b*_) for the red channel. Our method was illustrated using the usual subtraction (*r*_*f *_- *r*_*b*_). Although our method starts with a most common approach based on the usual subtraction, it can be applied to any other models for defining spot intensity *r *= f(*r*_*f*_,*r*_*b*_), where *r *is the signal intensity, as long as a background intensity is available. Our method can be applicable as long as the image analysis provides the signal intensity and background intensity. For example, our method using *Y*_2 _builds on the relationship between *r *= f(*r*_*f*_,*r*_*b*_) and *r*_*b*_.

**Figure 7:**
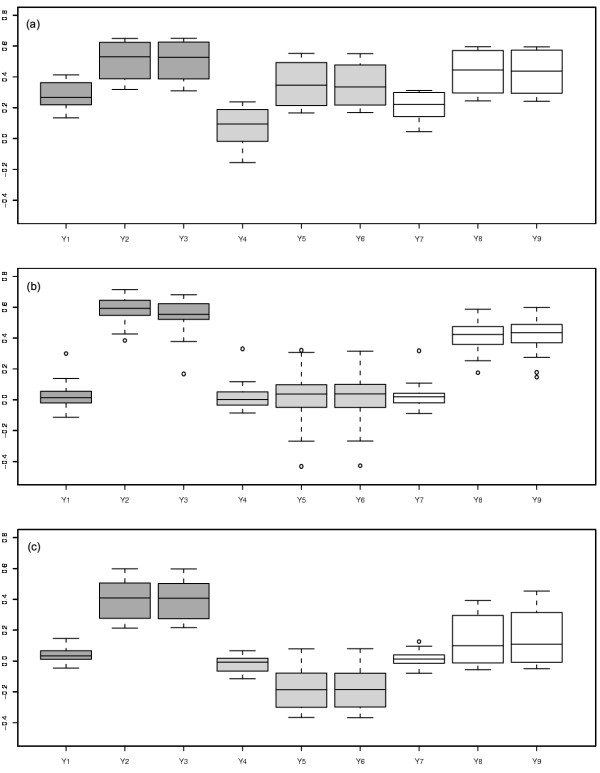
Boxplots of correlation coefficients between *M *and *Y*_*k *_using all slides. (a) Colorectal cancer data set from NCI 60, (b) Cortical Stem Cells Data (Park *et al. *[17]), (c) Kidney data (Pritchard *et al. *[18]). Dark grey boxplots are the distribution of correlation coefficients between *M *and the background measures from red channel (*Y*_*1*_, *Y*_*2*_, *Y*_*3*_), light grey boxplots are the corresponding correlation coefficients between *M *and the background measures from green channel (*Y*_*4*_, *Y*_*5*_, *Y*_*6*_), and white boxplots are those from both channels (*Y*_*7*_, *Y*_*8*_, *Y*_*9*_).

We recommend that experimentalists examine their data carefully and consider applying the two-stage normalization methods using *Y*_2 _and *Y*_3_. The performance of the two-stage normalization method tends to depend on the correlations between background measures and *M*. That is, if there is a strong relationship between them, our method has a large effect. Thus, for the experimentalists it might be important to tell when to use the two-stage normalization method. In order to answer this question, we compute correlations between *M *and all background measures from *Y*_1 _to *Y*_9_. Figure 7 shows the boxplots of correlation coefficients between *M *and *Y*_*k*_s for each data set. For example, Figure 7(a) shows the distribution of correlation coefficients from the NCI 60 data set. The correlation coefficients were computed for all ten slides. As expected, the correlations of *Y*_2 _and *Y*_3 _are relatively higher than those from other background measures. We also think that is why *Y*_2 _and *Y*_3 _have better performances than the other background measures.

Figure 7 also shows that the median values of correlations of *Y*_2 _and *Y*_3 _are higher than 0.5 for both CCD and SCD, while those for KD are smaller than 0.5. Note that CCD and SCD have more reduction in variability than KD in the two-stage normalization. For lower correlations of *Y*_*k*_s do not reduce variability much compared to the usual LOWESS normalization.

## References

[B1] Kerr MK, Martin M, Churchill GA (2000). Analysis of variance for gene expression microarray data. J Comput Biol.

[B2] Kerr MK, Martin M, Churchill GA (2001). Experimental design for gene expression microarrays. Biostatics.

[B3] Wolfinger RD, Gibson G, Wolfinger ED, Bennett L, Hamadeh H, Bushel P, Afshari C, Paules RS (2001). Assessing gene significance from cDNA microarray expression data via mixed models. J Comput Biol.

[B4] Schadt EE, Li C, Ellis B, Wong WH (2001). Feature extraction and normalization algorithms for high-density oligonucleotide gene expression array data. J Cell Biochem Suppl.

[B5] Kepler TB, Crosby L, Morgan KT (2002). Normalization and analysis of DNA microarray data by self-consistency and local regression. Genome Biology.

[B6] Yang YH, Dudoit S, Luu DM, Peng V, Ngai J, Speed TP (2002). Normalization for cDNA microarray data: a robust composite method addressing single and multiple slide systematic variation. Nucleic Acids Research.

[B7] Cleveland WS (1974). Robust locally weighted regression and smoothing scatterplots. Journal of the American Statistical Association.

[B8] Workman C, Jensen LJ, Jarmer H, Berka R, Gautier L, Nielser HB, Saxild HH, Nielsen C, Brunak S, Knudsen S (2002). A new non-linear normalization method for reducing variability in DNA microarray experiments. Genome Biol.

[B9] Wang Y, Lu J, Lee R, Gu Z, Clarke R (2002). Iterative normalization of cDNA microarray data. IEEE Trans Inf Technol Biomed.

[B10] Chen YJ, Kodell R, Sistare F, Thompson KL, Morris S, Chen JJ (2003). Normalization methods for analysis of microarray gene-expression data. J Biopharm Stat.

[B11] Yang MC, Ruan QG, Yang JJ, Eckenrode S, Wu S, McIndoe RA, She JX (2001). A statistical method for flagging weak spots improves normalization and ratio estimates in microarrays. Physiol Genomics.

[B12] Kim JH, Kim HY, Lee YS (2001). A novel method using edge detection for signal extraction from cDNA microarray image analysis. Exp Mol Med.

[B13] Kim JH, Shin DM, Lee YS (2002). Effect of local background intensities in the normalization of cDNA microarray data with a skewed expression profiles. Exp Mol Med.

[B14] Kooperberg C, Fazzio TG, Delrow JJ, Tsukiyama T (2002). Improved background correction for spotted DNA microarrays. J Comput Biol.

[B15] Edwards D (2003). Non-linear normalization and background correction in one-channel cDNA microarray studies. Bioinformatics.

[B16] Zhou Y, Gwadry FG, Reinhold WC, Miller LD, Smith LH, Scherf U, Liu ET, Kohn KW, Pommier Y, Weinstein JN (2002). Transcriptional Regulation of Mitotic Genes by Camptothecin-induced DNA Damage: Microarray Analysis of Dose- and Time-dependent Effects. Cancer Research.

[B17] Park T, Yi SG, Lee S, Lee SY, Yoo DH, Ahn Jl, Lee YS (2003). Statistical tests for identifying differentially expressed genes in time course microarray experiments. Bioinformatics.

[B18] Pritchard CC, Hsu L, Delrow J, Nelson PS (2001). Project normal: Defining normal variance in mouse gene expression. PNAS.

[B19] Weinstein JN, Myers TG, O'Connor PM, Friend SH, Fornace AJ, Kohn KW, Fojo T, Bates SE, Rubinstein LV, Anderson NL, Buolamwini JK, van Osdol WW, Monks AP, Scudiero DA, Sausville EA, Zaharevitz DW, Bunow B, Viswanadhan VN, Johnson GS, Wittes RE, Paull KD (1997). An information-intensive approach to the molecular pharmacology of cancer. Science.

[B20] Park T, Yi SG, Kang SH, Lee S, Lee YS, Simon R (2003). Evaluation of normalization methods for microarray data. BMC Bioinformatics.

